# Role of FDG-PET scans in staging, response assessment, and follow-up care for non-small cell lung cancer

**DOI:** 10.3389/fonc.2012.00208

**Published:** 2013-01-03

**Authors:** John Cuaron, Mark Dunphy, Andreas Rimner

**Affiliations:** ^1^Department of Radiation Oncology, Memorial Sloan-Kettering Cancer CenterNew York, NY, USA; ^2^Department of Nuclear Medicine, Memorial Sloan-Kettering Cancer CenterNew York, NY, USA

**Keywords:** PET, non-small cell lung cancer, staging, response assessment, follow-up

## Abstract

The integral role of positron-emission tomography (PET) using the glucose analog tracer fluorine-18 fluorodeoxyglucose (FDG) in the staging of non-small cell lung cancer (NSCLC) is well established. Evidence is emerging for the role of PET in response assessment to neoadjuvant therapy, combined-modality therapy, and early detection of recurrence. Here, we review the current literature on these aspects of PET in the management of NSCLC. FDG-PET, particularly integrated ^18^F-FDG-PET/CT, scans have become a standard test in the staging of local tumor extent, mediastinal lymph node involvement, and distant metastatic disease in NSCLC. ^18^F-FDG-PET sensitivity is generally superior to computed tomography (CT) scans alone. Local tumor extent and T stage can be more accurately determined with FDG-PET in certain cases, especially in areas of post-obstructive atelectasis or low CT density variation. FDG-PET sensitivity is decreased in tumors <1 cm, at least in part due to respiratory motion. False-negative results can occur in areas of low tumor burden, e.g., small lymph nodes or ground-glass opacities. ^18^F-FDG-PET-CT nodal staging is more accurate than CT alone, as hilar and mediastinal involvement is often detected first on ^18^F-FDG-PET scan when CT criteria for malignant involvement are not met. ^18^F-FDG-PET scans have widely replaced bone scintography for assessing distant metastases, except for the brain, which still warrants dedicated brain imaging. ^18^F-FDG uptake has also been shown to vary between histologies, with adenocarcinomas generally being less FDG avid than squamous cell carcinomas. ^18^F-FDG-PET scans are useful to detect recurrences, but are currently not recommended for routine follow-up. Typically, patients are followed with chest CT scans every 3–6 months, using ^18^F-FDG-PET to evaluate equivocal CT findings. As high ^18^F-FDG uptake can occur in infectious, inflammatory, and other non-neoplastic conditions, ^18^F-FDG-PET-positive findings require pathological confirmation in most cases. There is increased interest in the prognostic and predictive role of FDG-PET scans. Studies show that absence of metabolic response to neoadjuvant therapy correlates with poor pathologic response, and a favorable ^18^F-FDG-PET response appears to be associated with improved survival. Further work is underway to identify subsets of patients that might benefit individualized management based on FDG-PET.

## INTRODUCTION

Lung cancer is the leading cause of cancer death in the USA and worldwide. In 2009, there were an estimated 1,608,800 cases and 1,348,400 deaths due to lung cancer globally (Jemal et al., 2011), and it is estimated that there will be 226,160 new cases and 160,340 deaths attributable to lung cancer in the USA in 2012 (Siegel et al., 2012).

The management of non-small cell lung cancer (NSCLC) often requires a multimodality approach to accurately diagnose, stage, and treat patients. Some of the most important advances in the treatment of lung cancer have been the development and implementation of accurate and functional imaging. The advent of positron-emission tomography (PET) and computed tomography (CT) scanning has revolutionized the treatment of lung cancer, and each has become integral in the staging and treatment of NSCLC. The purpose of this article is to review the role of fluorodeoxyglucose (FDG)-PET scans in the diagnosis, staging, response assessment, and follow-up of patients treated for NSCLC. While several non-^18^F-FDG-PET radiotracers show promise in preclinical and early clinical evaluation, we have limited the scope of this review to ^18^F-FDG-PET as this is the most widely used, clinically relevant and extensively studied form of PET in the management of NSCLC.

## DIAGNOSIS AND EVALUATION OF LUNG LESIONS

In the primary evaluation of pulmonary lesions, FDG-PET scans are useful to distinguish between benign and malignant etiologies. Although numerous non-malignancy related conditions within the lungs take up FDG, including infection and inflammation, extensive work has been done in correlating FDG-PET positivity with pathological malignancy. A meta-analysis of studies investigating the accuracy of FDG-PET in diagnosing malignant pulmonary lesions estimated the sensitivity and specificity to be 96.8 and 77.8%, respectively (Gould et al., 2001). A separate meta-analysis found the sensitivity, specificity, and accuracy of ^18^F-FDG-PET in the diagnosis of lung lesions to be 96, 80, and 91% (Hellwig et al., 2001). In the same analysis, ^18^F-FDG-PET was superior to CT in the evaluation of nodal and distant metastasis, and changed therapeutic management in 18% of all of the cases studied.

Positron-emission tomography is particularly useful in differentiating benign from malignant obstructions in the setting of atelectasis. A retrospective study of 84 patients demonstrated a significantly higher sensitivity with ^18^F-FDG-PET/CT compared with CT alone in the detection of atelectasis-associated malignant lesions (91 vs 48%, *p* < 0.001; Cho et al., 2011). The authors concluded that, because FDG uptake was significantly higher in malignant lesions, PET can potentially reduce the number of unnecessary invasive procedures and more accurately select those patients with atelectasis that require further workup.

**FIGURE 1:**
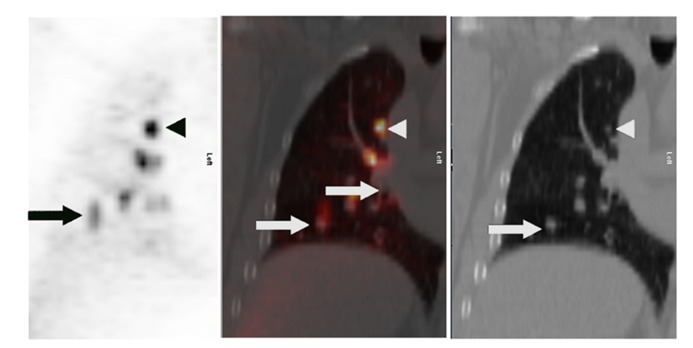
**Lung cancer patient with multiple right lung nodules**. Shown are corresponding PET (*left*), CT (*right*), and fusion (*middle*) images of a single coronal plane through the right lung. In the upper lobe, where respiratory motion is least, a punctate nodule (white arrowheads) demonstrates intense FDG uptake with a distinctly focal appearance (black arrowhead). In the lower region of the lung, where respiratory motion is greater, the FDG uptake of larger nodules (white arrows) appears relatively less-intense – probably due to “spreading” of the activity over a spatial volume during each breathing cycle, a kind of respiratory artifact. This “spreading” is most visually evident closest to the diaphragm (black arrow).

Positron-emission tomography has been shown to be less sensitive for the characterization of smaller lung lesions. This may be at least in part due to respiratory motion, which artificially decreases the FDG signal (**Figure 1**). Motion artifact can cause a significant underestimation of ^18^F-FDG uptake, which is commonly quantified with a parameter called the maximum standardized uptake value, or SUV_max_ (Liu et al., 2009).

**FIGURE 2:**
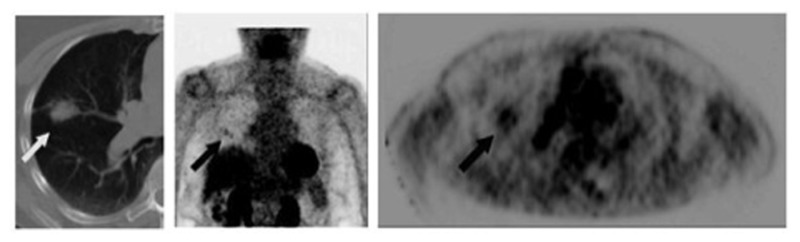
**Eighty-two-year-old female with growing right lung nodule**. Prior lung CT scans over a 1-year period had demonstrated increasing size of a spiculated-appearing right lung nodule (white arrow). FDG-PET/CT was performed for nodule characterization. Images show corresponding transaxial planes through the chest from the CT and FDG PET (*upper right*); and a 3D maximum intensity projection imaged centered on the chest region (*center image*). PET showed detectable, albeit minimal, activity in the right lung nodule (black arrows; SUV_max_ 2.1) and focal activity in the right pulmonary hilum (arrowhead) thought to represent lymph node FDG uptake. Wedge resection was performed; pathology found focal bronchopneumonia/granuloma.

Toba et al. (2010) showed the positive predictive value (PPV) of FDG-PET to be significantly lower with lesions <1 cm in size compared with larger lesions (0.36 vs 0.90, *p* = 0.015). The lower PPV of smaller lesions reflects a higher rate of falsely positive FDG-PET scans. An example of a false-positive lung nodule is shown in **Figure 2**.

In addition to the evaluation of primary lung lesions and the determination of malignant involvement, PET may also offer insight into the histology of the imaged malignancy. Correlations of pathology with the SUV_max_ of tumors on preoperative imaging have shown bronchioalveolar carcinoma and well-differentiated tumors to be less ^18^F-FDG-avid (Vesselle et al., 2008) and squamous cell carcinomas to have a consistently higher ^18^F-FDG uptake compared with other histologies (Aquino et al., 2007; de Geus-Oei et al., 2007). This additional characterization of lesions by PET may be able to distinguish synchronous primary tumors from metastatic disease, and offer prognostic information beyond what is gathered with CT-based imaging.

## STAGING OF THE MEDIASTINUM

Beyond the evaluation of the primary tumor, the accurate staging of the mediastinal lymph nodes is critically important in the management of NSCLC. The presence of mediastinal lymph node involvement changes the management approach to multimodality therapy including chemotherapy, surgery, and radiation therapy, while early-stage node-negative lung cancer is usually treated with a single local treatment modality focusing on the primary parenchymal lung lesion only, such as surgical resection or stereotactic body radiation therapy (SBRT).

Numerous retrospective studies have investigated the ability of PET to detect lymph node metastases. A single institutional comparison of CT and ^18^F-FDG-PET/CT staging of the mediastinum in relation to pathological findings showed ^18^F-FDG-PET/CT to have superior sensitivity, specificity, accuracy, positive and negative predictive value (Yang et al., 2008). Li et al. (2012) reported an excellent negative predictive value of ^18^F-FDG-PET (91%) in the evaluation of early stage T1-2N0 tumors. A meta-analysis of 39 studies found superior sensitivity and specificity for lymph node involvement based on ^18^F-FDG-PET when compared with CT. Sensitivity and specificity were 85 and 90%, for ^18^F-FDG-PET, and 61 and 79% for CT, respectively (Gould et al., 2003). Another meta-analysis (Lv et al., 2011) confirmed the excellent specificity of FDG-PET in the staging of the mediastinum (95%), although the authors reported a lower sensitivity (68%) than other comparable studies.

The comparison between PET and CT has also been investigated in the prospective setting. Scott et al. (1996) enrolled 27 patients with known or suspected NSCLC to undergo both a CT and ^18^F-FDG-PET scan, and compared the modalities in their ability to detect N2 and N3 metastasis, which was assessed by surgical confirmation. CT had a sensitivity of 60%, a specificity of 93%, and a PPV of 60%, whereas ^18^F-FDG-PET in conjunction with CT was 100% sensitive, 98% specific, and had a PPV of 91%. A similarly designed, larger study from Belgium confirmed the superiority of ^18^F-FDG-PET added to CT in the accurate staging of N2/N3 metastasis (Vansteenkiste et al., 1998). Pieterman et al. (2000) reported a sensitivity and specificity of 91 and 86% for ^18^F-FDG-PET compared with 75 and 66% for CT. A multicenter prospective investigation from Japan similarly demonstrated a statistically significant improvement in the accuracy and specificity of integrated ^18^F-FDG-PET/CT with a modern hybrid scanner over standard CT alone in the evaluation of mediastinal lymph nodes (Kubota et al., 2011). A summary of the studies that have evaluated the performance of ^18^F-FDG-PET and ^18^F-FDG-PET/CT in evaluating the mediastinum is shown in **Table 1**.

**Table 1:** Performance of PET and PET/CT in detection of mediastinal lymph node metastasis.

	No. of patients	Sensitivity (%)	Specificity (%)	Accuracy (%)	Positive predictive value (%)	Negative predictive value (%)
Scott et al. (1996)	27	100	98	–	91	–
Vansteenkiste et al. (1998)	68	93	95	94	–	–
Pieterman et al. (2000)	102	91	86	–	–	–
Gould et al. (2003)	3078	85	90	–	–	–
Yang et al. (2008)	122	86	85	85	64	95
Lv et al. (2011)	2550	68	95	–	–	–
Kubota et al. (2011)	81	87.2	72.5	77.8	–	–
Li et al. (2012)	200	44	83	78	29	91

An interesting caveat to the superior performance of ^18^F-FDG-PET/CT in the evaluation of the mediastinum was reported by a study from Ireland (Al-Sarraf et al., 2008). In their retrospective review of 206 patients and 1145 lymph nodes, the authors stratified lymph nodes by size and found that ^18^F-FDG-PET/CT had a significantly lower specificity and accuracy for nodes >1 cm as compared to <1 cm, illustrating the higher possibility of false positivity with large lesions. Importantly, ^18^F-FDG-PET/CT for large lymph nodes still performed better than CT alone. These findings were consistent with Shiraki et al. (2004), who reported that false-positive nodes were significantly larger than true-negative nodes as staged by FDG-PET. These studies illustrate an important limitation of FDG-PET/CT, and reaffirm that FDG-PET-positive lymph nodes still require pathological confirmation by mediastinoscopy.

## DETECTION OF DISTANT METASTASIS

The need for accurate staging of patients with NSCLC applies not only to the mediastinum but also to the evaluation for distant metastatic disease. Historically, bone scintigraphy had been a mainstay in the initial evaluation of NSCLC due to the tumor’s propensity to spread to the bones. Recently, several efforts have shown that ^18^F-FDG-PET/CT offers superior rates of detection and has obviated the need for bone scans at presentation.

^18^F-FDG-PET/CT is particularly effective at detecting bone metastasis, with one study reporting a sensitivity, specificity, and accuracy of 93.9, 98.9, and 97.8% (Liu et al., 2010). A comparison of technetium 99m-methylene diphosphonate (^99m^Tc-MDP) bone scintigraphy to ^18^F-FDG-PET/CT was first described by Bury et al. (1998), who showed a higher accuracy with ^18^F-FDG-PET in the detection of osseous metastasis. These findings have been corroborated by other reports (Hsia et al., 2002; Gayed et al., 2003; Cheran et al., 2004). A German study of lung cancer patients compared integrated ^18^F-FDG-PET/CT versus two types of bone scans – a standard two-dimensional “planar” bone scan with ^99m^Tc-MDP and a three-dimensional bone PET scan with the bone-tracer fluorine-18 fluoride (^18^F-PET) – a different tracer from ^1^^8^F-FDG-PET that has become increasingly available worldwide. The investigators reported that ^18^F-FDG-PET/CT performed with a lower sensitivity than the ^18^F-PET bone scan, but was superior to conventional ^99m^Tc-MDP bone scintigraphy (Kruger et al., 2009). However, neither of the bone tracers – ^18^F-PET nor ^99m^Tc-MDP scintigraphy – can distinguish viable tumor from treated disease, whereas the metabolism tracer ^18^F-FDG-PET is much more specific for viable tumor. Even when combined with testing of alkaline phosphatase levels, which is often elevated in patients with bone metastasis, ^99m^Tc-MDP bone scan still performs inferiorly to ^18^F-FDG-PET/CT (Min et al., 2009). An important limitation to the ability of FDG-PET to detect neoplastic disease is the potential for false positivity, as numerous benign conditions (including trauma, infection, and physiological variants) are associated with a substantial amount of radiotracer uptake. The question was most thoroughly answered by a recent meta-analysis of 17 studies comparing ^18^F-FDG-PET/CT, ^18^F-FDG-PET, MRI, and bone scintography. The pooled sensitivity of each of the modalities in the detection of metastasis was 92, 87, 77, and 86%; the specificity was 98, 94, 92, and 88%, respectively. When analyzed by diagnostic odds ratio, ^18^F-FDG-PET/CT (2014.9) was significantly superior to ^18^F-FDG-PET (75.26), MRI (161.2), and bone scintography (37.85; Qu et al., 2012).

FDG-PET is also accurate overall in the detection of extra-osseous distant metastases. In the same study demonstrating the superiority of ^18^F-FDG-PET in staging of the mediastinum, Pieterman et al. (2000) reported on the high sensitivity and specificity of ^18^F-FDG-PET in the detection of distant metastases alone (92 and 83%), and 11% of patients in their study had distant metastases detected by ^18^F-FDG-PET that other modalities had failed to detect. The use of PET appears to be particularly important in the workup of patients with locally advanced NSCLC who may be offered curative therapy, as one study reported 24% of patients with clinical stage III cancer had previously undetected distant metastases when evaluated by ^18^F-FDG-PET (Mac Manus et al., 2001). This phenomenon of stage migration has been well documented (Chee et al., 2008; Farjah et al., 2009; Dinan et al., 2012) and most likely contributes to the association of ^18^F-FDG-PET with improved overall survival in patients with NSCLC.

In the characterization of adrenal lesions found by CT or MRI, ^18^F-FDG-PET has excellent sensitivity, specificity, and accuracy (100, 94, 96%, respectively; Yun et al., 2001); however, an FDG-avid adrenal nodule can represent a hyper-functioning adenoma. If the finding of an FDG-avid adrenal lesion potentially impacts patient care, then further characterization of the adrenal lesion should be considered, including the use of biochemical assays of adrenal function and/or adrenal-protocol CT or MRI studies and pathologic confirmation.

Because of the high background ^18^F-FDG uptake of normal brain parenchyma, the ability of PET to detect brain metastasis is limited. When compared with other imaging modalities, ^18^F-FDG-PET appears to offer no additional information regarding the presence of metastatic disease in the brain (Palm et al., 1999; Posther et al., 2006). The current standard of care for all patients with clinical stage ≥ IB is to evaluate the brain with a dedicated MRI of the brain (Ettinger et al., 2010), and there is currently no indication that ^18^F-FDG-PET/CT will be able to replace this diagnostic test.

## PREDICTION AND EVALUATION OF RESPONSE

Beyond the initial staging, ^18^F-FDG-PET may be useful in the prediction of response to therapy. By offering information regarding metabolic activity in addition to structural appearance, ^18^F-FDG-PET can more accurately characterize lesions prior to therapy. Furthermore, due to treatment effects after neoadjuvant chemotherapy and radiation therapy, including shrinkage of disease burden and radiation-induced inflammation and fibrosis, CT is limited in its ability to accurately predict prognosis after initial therapy, whereas ^18^F-FDG-PET may offer a more accurate evaluation of initial response.

### PREDICTION OF RESPONSE AFTER SBRT FOR EARLY-STAGE NSCLC

For early stage tumors, SBRT has been widely adopted as an alternative to surgical resection in medically inoperable patients. There are mixed data regarding the utility of pretreatment ^18^F-FDG-PET scans in the prediction of response to SBRT. Clarke et al. (2012) retrospectively showed that higher pretreatment SUV_max_ was associated with worse recurrence-free survival and a higher rate of distant failure in patients with stage I lung cancer treated with SBRT. A larger study by Takeda et al. (2011) showed significantly higher local control rates for lower SUV_max_ in patients with localized, node-negative lung cancer treated with SBRT.

In contrast, a similar study with a shorter median follow-up of 16.9 months showed no significant correlation with SUV_max_ and mediastinal failure, distant failure, or overall survival (Burdick et al., 2010). A study from Indiana University with a much longer follow-up of 42.5 months also showed no correlation between pretreatment SUV_max_ and local control or overall survival, but the pretreatment SUV_max_ was only available for 55% of the patients studied (Hoopes et al., 2007). A prospective study evaluated 39 tumors treated with SBRT and followed with CT and ^18^F-FDG-PET, and while both imaging modalities were useful in evaluating local radiographic and metabolic response, further analysis is needed to correlate these responses with long term treatment outcomes (Mohammed et al., 2011).

### PREDICTION OF RESPONSE AFTER DEFINITIVE SEQUENTIAL OR CONCURRENT CHEMORADIATION IN INOPERABLE PATIENTS

For patients with unresectable disease treated with chemotherapy or chemoradiation, studies evaluating the prognostic value of pretreatment ^18^F-FDG-PET scans are limited by heterogenous patient populations that are ultimately treated with a wide range of modalities. A meta-analysis of 13 studies and 1474 patients showed high pretreatment SUV to be a statistically significantly poor prognostic factor for survival; however, the majority of the studies included patients with any stage (I–IV) disease, and SUV threshold determinations were often arbitrary and differed among authors (Berghmans et al., 2008). A slightly more homogenous population was retrospectively investigated by Hoang et al. (2008) who demonstrated no significant correlation between pretreatment ^18^F-FDG uptake and survival among 214 patients with stage III–IV NSCLC, the majority of whom were not treated with surgery.

Post-treatment ^18^F-FDG-PET and CT have been compared in a prospective manner in patients treated with definitive radiation or chemoradiation, and ^18^F-FDG-PET response was found to be more significantly correlated with survival than response as assessed by CT (Mac Manus et al., 2003). Other studies have confirmed a poorer prognosis in patients with a higher volume of residual metabolically active tumor after definitive treatment (Lee et al., 2012).

Induction chemotherapy (IC) can be used as neoadjuvant therapy prior to consolidative radiotherapy. Decoster et al. (2008) studied 31 patients that were treated with IC for unresectable stage III disease. Patients received a baseline ^18^F-FDG-PET and CT scan, and scans were repeated after three cycles of IC. Response by FDG-PET and by CT was correlated with time to progression and overall survival. The authors showed that a complete response (CR) by ^18^F-FDG-PET after IC is associated with a significantly longer time to progression (*p* = 0.026) and overall survival (*p* = 0.004) than non-CRs, and those with a partial response had a longer time to progression but only a trend toward longer survival compared with non-responders. These findings demonstrate the ability of ^18^F-FDG-PET to offer accurate prognostic and predictive information for patients undergoing IC that cannot undergo surgical resection.

### INDUCTION CHEMOTHERAPY PRIOR TO PREOPERATIVE CHEMORADIATION THERAPY

In selected patients, there may be a role for neoadjuvant therapy for locally advanced, potentially resectable NSCLC. While the optimal regimen in this setting is unclear, two commonly applied options include IC followed by surgery, or preoperative chemoradiation and surgery. Investigators have shown ^18^F-FDG-PET to be a useful predictor of both pathological and clinical response in patients treated with IC prior to preoperative conformal radiotherapy (CRT). A German study showed that the percentage decrease in SUV_max_ on serial ^18^F-FDG-PET scans obtained for initial staging, after IC, and after CRT, was significantly correlated with histopathological response in patients that ultimately underwent surgical resection (Pottgen et al., 2006). In another study, patients that underwent a similar sequence of treatment were evaluated with both a staging ^18^F-FDG-PET and a restaging ^18^F-FDG-PET after the completion of IC and CRT. Survival was significantly longer for patients who experienced a reduction of >80% of the original average SUV, defined as the (SUV_max_ + SUV of surrounding background structures)/2. Those patients who had only a partial response or progressive disease had a significantly worse outcome (Eschmann et al., 2007). These results were confirmed by Choi et al. (2002) who showed residual ^18^F-FDG uptake after preoperative chemoradiation therapy to be significantly correlated with the degree of pathological response.

Several non-^18^F-FDG-PET radiotracers show promise in the evaluation of treatment response, due to their ability to characterize unique aspects of tumor behavior such as DNA synthesis, hypoxia, amino acid use, and hormone receptor expression (Dunphy and Lewis, 2009). In conjunction with information about glucose metabolism offered by FDG-based PET, these tracers may offer more comprehensive and specific information about tumor characteristics and predicted response to therapy in the future.

In summary, the ability of FDG-PET to offer predictive and prognostic information regarding the response to initial therapy is well documented, and can aid in the proper selection of patients that are most likely to benefit from further therapy in the form of surgery or radiation.

## FOLLOW-UP AND SURVEILLANCE

**FIGURE 3:**
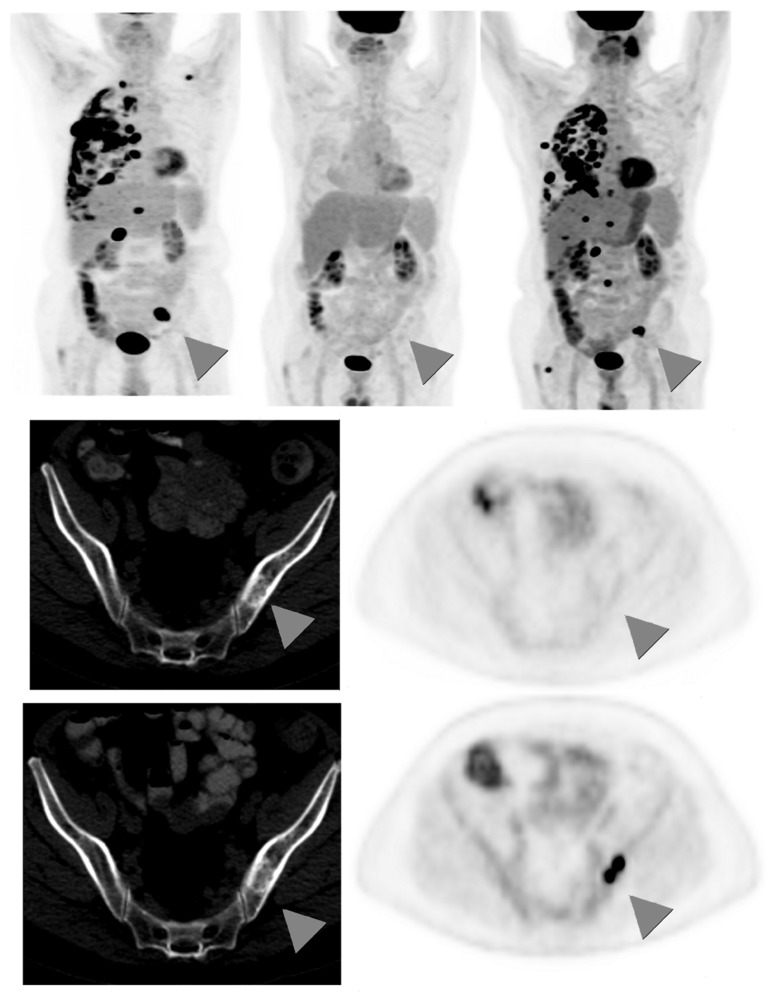
**Sixty-seven-year-old male with stage IV non-small cell lung cancer**. *Top* *row*: 3D maximum intensity projection (MIP) FDG-PET images obtained at baseline (*left*); after 4 months treatment with pemetrexed and bevacizumab (*middle*); and 9 months after the baseline scan, switched to second-line docetaxel therapy due to progression on interim scans (*right*). Arrowheads**point to an FDG-avid osseous metastasis in the left ilium that initially disappears in response to treatment but then recurs. *Middle row*: corresponding CT (*left*) and PET (*right*) images of a single transaxial plane through the pelvis, showing the lesion of the left ilium on CT that showed no FDG-avidity after 4 months treatment with pemetrexed and bevacizumab, suggesting a treated disease (arrowheads). *Bottom row*: the subsequent scan, 9 months after baseline, showed no change in the CT appearance of the left iliac bone lesion, but the lesion now demonstrates intense FDG uptake, consistent with a viable/recurrent metastasis.

There is limited data regarding the role of ^18^F-FDG-PET in the long-term follow-up of patients treated with definitive therapy for NSCLC, and routine surveillance PET scans are not recommended (Ettinger et al., 2010). However, many benign conditions, including atelectasis, consolidation, and radiation fibrosis, are difficult to distinguish from locoregional recurrence on standard CT imaging (Lever et al., 1984). In these settings, ^18^F-FDG-PET is useful in not only accurately identifying true malignant relapse but also offering prognostic information. Hicks et al. (2001) reported ^18^F-FDG-PET’s sensitivity for relapse as 98%, specificity as 82%, and overall accuracy as 93%, with ^18^F-FDG-PET negativity being highly predictive of better survival. When feasible, PET-positive areas should be corroborated with pathological analysis, as benign conditions including inflammation and fibrosis can exhibit hypermetabolic activity. An example of recurrence of distant metastasis as seen by ^18^F-FDG-PET is shown in **Figure 3**.

FDG-PET is useful for detecting recurrence after SBRT, as higher SUVs on scans obtained more than 6 months after treatment have been shown to be associated with higher local recurrence rate (Zhang et al., 2012). An important finding in this study was that treated areas may remain persistently hypermetabolic, leading to the inability to detect local recurrence; SUVs from ^1^^8^F-FDG-PET scans done within 6 months of treatment were not correlated with local recurrence. A similar phenomenon was observed in the study by Hoopes et al. (2007), who reported that several patients had moderately hypermetabolic activity but no evidence of local, nodal, or distant recurrence as seen on scans performed 2 years after treatment. This persistent uptake may be due to the unique reaction within tissue and tumor after SBRT, including more persistent inflammation and fibrosis compared with conventional fractionation. As the use of this modality continues to increase, further study is warranted to investigate the role of FDG-PET in the evaluation, treatment, and follow-up of patients treated with SBRT.

## CONCLUSION

FDG-PET, particularly current state-of-the-art FDG-PET/CT, plays an instrumental role in all phases of the management of lung cancer offering superior accuracy in the diagnosis of lung tumors, detection of nodal and distant metastasis, and delineation of T stage compared with other modalities. Importantly, ^18^F-FDG-PET is less accurate with lesions <1 cm in the lung and lesions that exhibit significant motion. While still superior in accuracy to other modalities overall, these limitations warrant further investigation of sub-centimeter, ^18^F-FDG-PET-negative lesions, in most cases. Pathological evaluation of ^18^F-FDG-PET-positive lesions is necessary given the possibility for false-positive findings. FDG-PET scans also offer predictive and prognostic information after both neoadjuvant and definitive therapy and are useful in the workup of suspected recurrences. The increased use of FDG-PET scans will contribute to the more accurate selection of patients for appropriate treatment, which may improve outcomes and help avoid toxic therapies that are unlikely to provide benefit to patients.

## Conflict of Interest Statement

The authors declare that the research was conducted in the absence of any commercial or financial relationships that could be construed as a potential conflict of interest.
